# Effect of Dietary Modification and Physical Activity on Obese Young Adults Going to Gym for Weight Loss in Central India: A Before and After Study

**DOI:** 10.7759/cureus.40832

**Published:** 2023-06-22

**Authors:** Utkarsha P Karmore, Ujwala U Ukey, Sarita K Sharma

**Affiliations:** 1 Community Medicine, Government Medical College Nagpur, Nagpur, IND; 2 Community Medicine, Government Medical College and Hospital, Nagpur, IND

**Keywords:** gym, obese young adults, before and after study, physical activity, dietary modification

## Abstract

Background: An adequate diet is a prerequisite for appropriate growth and development so as to remain active. Balanced nutrition coupled with physical activity forms a healthy lifestyle which eventually leads to multiple health benefits such as positive mental health and a lower risk of noncommunicable diseases like diabetes, hypertension, etc. It has become an upcoming trend for young adults to join a gym. In order to maintain weight members, go to the gym because they consider it as a positive opportunity to boost self-esteem and to make appropriate health decisions to feel better. There is a need to highlight with the help of research studies that lifestyle modification in the form of diet and physical activity on a regular basis can help in controlling obesity.

Methods: A before and after the study was carried out in Nagpur city located in Central India for a period of 8 months (April 1, 2022 to November 30, 2022). The study subjects were obese young adults going to the gym in Nagpur city. Diet and physical activity interventions were given for a duration of three months to the study subjects. The study instrument was a predesigned and pretested questionnaire.

Results: In the present study, total 110 study subjects responded by completely filling out the questionnaire The total calorie and protein intake of the study subjects before and after giving intervention for three months was found to be highly significant (p-value < 0.0001). The change in mean anthropometric parameters of the study subjects before and after giving intervention for three months was found to be highly significant (p-value < 0.0001).

Conclusions: Dietary modification combined with physical activity for an average of 75 minutes is the most effective short-term intervention for weight loss. The present study concludes that lifestyle modifications can reverse the trend of obesity. It is reversible, and obese individuals can normalize their BMI with appropriate interventions as was performed with the present obese study subjects.

## Introduction

An adequate diet is essential from the very early stages of life for appropriate growth and development so as to remain active [1]. Balanced nutrition coupled with physical activity form a healthy lifestyle which eventually leads to multiple health benefits such as positive mental health and a lower risk of noncommunicable diseases like diabetes, hypertension, etc. [2,3].

People may settle for food items that are higher in calories and lower in nutritional value when healthy options are not available. To prevent the adverse health effects this might cause, creating and supporting an environment that advocates healthy food is an important part of public health practices [4]. It is essential to address nutrition in terms of foods, rather than only nutrients so that the consuming general population follows it correctly [1]. A balance in the calories consumed from food and those burnt from physical activity is considered to be achieved when body weight does not change over a period of time. A considerable change in dietary patterns and physical activity level is needed to prevent obesity and maintain weight in the recommended range. Obesity is a disorder involving excessive body fat that increases the risk of health problems [5]. Many factors that contribute to obesity include genetics, eating pattern, physical activity levels, sleep routines, and certain medications [6].

Globally more than one billion adults are overweight and at least 300 million of them are clinically obese. Over recent years many reports have suggested that physical activity plays a positive role in health [2]. Often coexisting in developing countries with under-nutrition, obesity has also become prevalent with serious adverse impacts on health dimensions, affecting virtually all ages and socioeconomic groups. India is one of the dual-burden countries wherein undernutrition decreases and overnutrition increases by about the same proportion [7]. As many as 6.4% of women and 4.0% of men are affected due to obesity. It is imperative to create awareness about this burden and to foster healthy lifestyle factors such as diet and exercise to reduce obesity and various health outcomes related to it [8].

It has become an upcoming trend for young adults to join a gym. In order to maintain weight members, go to the gym because they consider it as a positive opportunity to boost self-esteem and to make appropriate health decisions to feel better [9]. There is widespread ignorance and negligence with regard to controlled food intake and determination toward continuous physical activity. So, many youngsters who are not enough motivated join the gym and leave it without actually achieving their goals. This is probably more common with obese adults who want to reduce their weight but somehow are unable to do so because immediate effects on the reduction of weight are not visible to them. There is a need to highlight with the help of research studies that lifestyle modification in the form of diet and physical activity on a regular basis can go a long way in helping to control obesity.

A literature search reveals that there is not much data available on the effects of dietary modifications and physical activity on the maintenance of weight more so from the current study area. Against this backdrop, the current study was carried out with the objectives to determine the effect of dietary modification and physical activity on weight loss in obese young adults going to the gym.

## Materials and methods

Study design and study duration

A before and after study was carried out in Nagpur city located in Central India. The study was carried out for a period of eight months (April 1, 2022 to November 30, 2022).

Study population

The study subjects were obese young adults attending a gym in Nagpur city who fulfilled the required inclusion criteria.

*Inclusion Criteria* 

Freshly enrolled gym-going obese young adults aged 18 to 40 years whose body mass index (BMI) was more than 25 kg/m^2^ and waist circumference was above 80 centimeters (cm) [10]. BMI cut-offs for the Asian Pacific region given by WHO were followed in the present study [11]. Only those individuals who had registered with gym membership for the next three months with regular gym attendance were included in the study. Considering the feasibility criteria and the time required for dietary modification and physical activity to show their effect on weight reduction these inclusion criteria were decided.

*Exclusion Criteria* 

Subjects who were not willing to participate in the study were excluded from the study.

Sample size and sampling technique

It was estimated based on the effect of exercise on anthropometric parameters reported in the published article [12]. The minimum required sample size was found to be 98. The study involved a nonprobability sampling technique wherein the subjects were chosen as per convenience sampling from the gym. In this city, which is located in Central India, there are a total of 35 to 40 established gyms. A list of all the gyms was procured. The owners of the gym were contacted and one gym from which permission was obtained regarding the conduct of the study was selected by convenience sampling method.

Ethical considerations

Approval was obtained from the Institutional Ethical Committee (IEC) Government Medical College Nagpur, as per letter number 1675/EC/Pharmacology/GMC/Nagpur. Permission was obtained from the manager of the gym regarding the conduct of the study. The nature and purpose of the survey were explained to the study subjects. A written informed consent was obtained from study participants and due care was taken to maintain complete anonymity of the study participants.

Intervention

All the obese young adults were provided with nutritional education, and information about food choices, cooking, and eating habits. A gradual reduction of calorie intake by 500 calories per week was advised to avoid any nutritional deficiency or complication. In order to achieve this, an individual customized week-wise dietary plan was prepared for each participant based on their requirement. All participants were instructed to follow the plan meticulously. The dietary modification was done keeping in mind the probability of overeating during parties and holidays. Consumption of a high-protein diet was emphasized. The participants were encouraged to consume more cereals and pulse green leafy vegetables, salad, fruits, nuts, eggs, chicken, etc. as per their dietary preferences. Participants were instructed to consume sugar, and fats-oils sparingly and to replace refined flour and white bread with multi-grain flour, brown/wheat bread, and oats. This reduction of 500 calories was applicable to all till they achieve recommended calorie intake as per their targeted BMI.

Regarding physical activity, each subject went through a five days-weekly physical training program for 60-90 minutes (with an average of 75 minutes) per training session under the supervision of professional coaches. The distribution of 60-90 minutes of physical activity included a combination of aerobics, strength training, weightlifting, and cardio exercises. Different body area muscles were given importance on specific weekdays such as Monday upper body, Tuesday back, Wednesday lower body, Thursday chest, etc. All these interventions were given for a duration of three months (around 90 days) to the study subjects.

Data collection

The study instrument was a predesigned and pretested questionnaire with questions based on sociodemographic information like age, gender, religion, education, occupation, number of family members and monthly income of the family, dietary history, and anthropometric parameters. Data collection was carried out by face-to-face interviews which were conducted in the vernacular language of the study subjects that is Marathi and Hindi. The interview was conducted in the gym itself from 7am to 9am and 6pm to 9pm. Dietary history was obtained by 24 hours oral recall method at the time of enrollment (baseline) of the study subjects that is before initiation of the intervention and three months after the intervention. Anthropometric measurements such as height, weight, waist circumference, and hip circumference were obtained at the time of enrollment (baseline) of the study subjects that is before initiation of the intervention and three months after the intervention.

Data management was done after the completion of data collection. Data obtained from interviews was cleaned, decoded, and then entered into a Microsoft Excel spreadsheet. Tables and graphs were prepared using Microsoft Word and excel software. Statistical software STATA version 14.0 was used for data analysis. Continuous variables (height, weight, body mass index, waist circumference and hip circumference) were presented as mean ± standard deviation (SD). Categorical variables were expressed in frequency and percentages. Anthropometric parameters were compared before and after dietary modification and physical activity by performing paired t-test. P-values of less than 0.05 was considered statistically significant.

## Results

In the present study, out of total 110 study participants, 58 (52.73%) were male and 52 (47.27%) were female. The mean age of the study participants was 26.90 ± 3.65 years. Most of the study participants, i.e., 83(75.45%) were Hindu by religion. The socioeconomic status of the study participants was calculated by using a modified Kuppuswamy scale. Sixty-three (52.27%) study participants belong to upper middle socioeconomic status and 6 (5.45%) were from upper socioeconomic status. The details of socio-demographic factors are shown in Table 1.

**Table 1:** Distribution of study subjects according to sociodemographic factors.

Variables		Number of subjects	Percentage
Age (in years)	18-20	01	0.90
	21-25	40	36.37
	26-30	51	46.36
	31-35	16	14.56
	36-40	02	1.81
Gender	Male	58	52.73
	Female	52	47.27
Religion	Hindu	83	75.45
	Muslim	04	3.65
	Buddhist	13	11.81
	Christian	02	1.81
	Sikh	08	7.28
Socioeconomic status	Upper (I)	06	5.45
	Upper middle (II)	63	57.27
	Lower middle (III)	41	37.28
	Upper lower (IV)	00	00
	Lower (V)	00	00

During the data collection period of 90 days, the daily attendance of the study participants in the gym was noted. Table 2 reveals distribution of study participants according to gym attendance. Majority of the study participants attended gym for 70 to 79 days. The mean attendance of the study participants was 69.47 ± 3.82 days. The attendance in the gym during the study period was in the range of 60-77 days.

**Table 2:** Distribution of study subjects according to gym attendance.

Number of days	Number of subjects	Percentage
60-69	46	41.81
70-79	64	58.19

In the present study, the total calorie and protein intake of study participants was calculated by 24 hours oral dietary recall method. After applying paired t-test the difference in the total calorie and protein intake of the study subjects before and after giving intervention for three months was found to be highly significant (p-value < 0.0001). The t-value for total calorie intake is 48.6335 and t-value for total protein intake is 7.0726. The tabular representation of the same is represented in Table 3.

**Table 3:** Distribution of study subjects according to total calorie and protein intake by 24hrs dietary oral recall method. Abbreviations: SD - Standard deviation, HS - Highly significant, kcal - Kilocalories, g - gram, kg- kilogram

	Before (Mean ± SD)	After (Mean ± SD)	t-value	P-value
Total calorie intake (kcal/day)	4019.09 ± 399.88	2175.45 ± 233.52	48.6335	<0.0001, HS
Total protein intake (g/kg body weight)	0.94 ± 0.78	1.47 ± 0.11	7.0726	<0.0001, HS

Various anthropometric measurements such as height, body weight, waist circumference andhip circumference of the study participants were recorded. From these BMI and waist hip ratio was obtained. It was observed that height of the study participants was in the range of 1.50 to 1.89 meter with a mean value of 1.63 ± 0.06 meter. Table 4 shows change in mean anthropometric parameters of the study participants. After applying paired t-test the change in mean anthropometric parameters of the study subjects before and after giving intervention for three months was found to be highly significant (<0.0001).

**Table 4:** Change in mean anthropometric parameters of the study subjects. Abbreviations: SD - Standard deviation, HS - Highly significant, kg - kilogram, m - meter, cm - centimeter

Parameters	Before (Mean ± SD)	After (Mean ± SD)	t-value	P-value
Weight (kg)	73.83 ± 5.04	65.32 ± 4.46	29.2385	< 0.0001, HS
BMI (kg/m^2^)	27.82 ± 1.92	24.59 ± 1.19	27.0877	<0.0001, HS
Waist circumference (cm)	89.24 ± 5.46	83.40 ± 4.76	25.5581	<0.0001, HS
Hip circumference (cm)	98.34 ± 7.29	93.56 ± 5.96	14.7385	<0.0001, HS
Waist hip ratio	0.90 ± 0.04	0.89 ± 0.03	5.3768	<0.0001, HS

**Figure 1:**
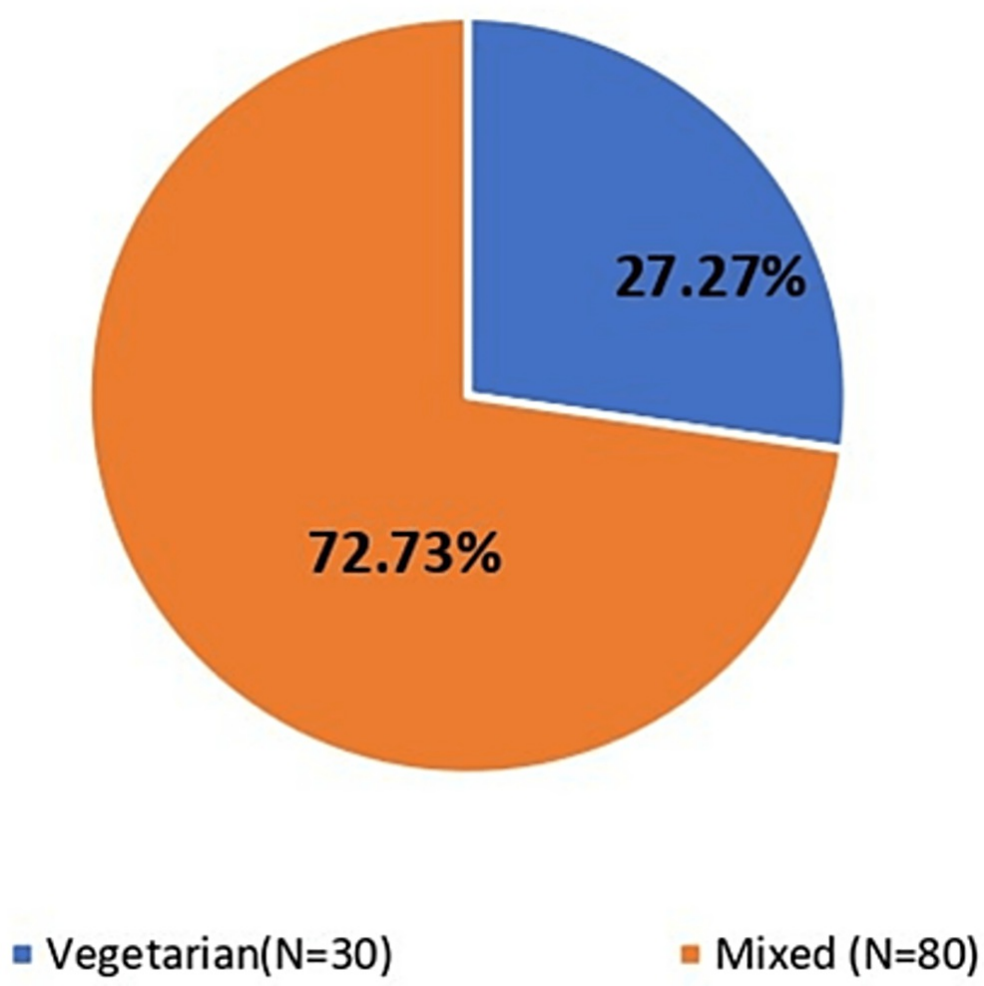
Distribution of study subjects according to type of diet.

In the present study, the diet pattern of the study participants was asked for in the form of whether vegetarian only or not. It was observed that most of the study subjects 80 (72.73%) were having mixed type of diet and 30 (27.27%) study participants were vegetarian. The graphical representation of the same is represented in Figure 1.

**Figure 2:**
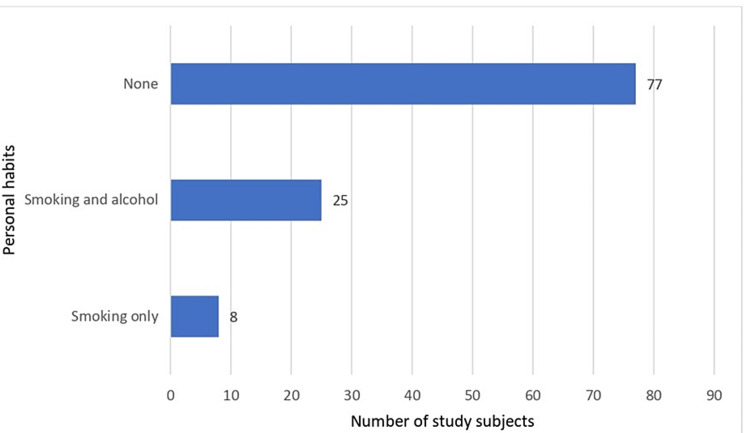
Distribution of study subjects according to personal habits.

Figure 2 shows distribution of study subjects according to personal habits. Most of the study subjects 77 (70.00%) were not having any habit of smoking and alcoholism and eight (7.2%) of study subjects were smokers.

## Discussion

Obesity has become a risk factor for major causes of death, including cardiovascular diseases, cancers, and diabetes. Obesity is linked with morbidity including osteoarthritis, gall bladder disease, sleep apnea, and respiratory impairment. It is also coupled with poor quality of life, including diminished mobility and social disapproval. Considering the alarming rise in overweight and obesity in developed as well as developing countries, the situation needs urgent attention [13,14]. Therefore the present study focused on the control of obesity in young adults by modifying two vital aspects of energy balance which include energy intake and energy expenditure.

Energy intake was assessed by daily dietary consumption and energy expenditure in the form of physical activity. In energy intake, the intervention comprised education about adherence to a low energy density diet, greater intake of fruits, vegetables, and whole grains, and decreased sugar consumption helped study participants in weight loss. In addition, portion control, taking regular breakfast, increased daily water intake, and studying food labels for calorie content while shopping for groceries facilitated weight loss. And energy expenditure was related to education about increased physical activity like regular walking, jogging, and aerobic exercises such as swimming, cycling, etc. for an average of 75 minutes.

In the present study, more than half of the study participants had a gym attendance of over 70 days during 90 days of intervention. The minimum number of days attended was 60. Overall good attendance is indicating that the intervention was received for time enough to show its effect. A greater improvement was seen in those who are engaged in both physical activity and healthy eating with dietary modifications.

It was found that the daily calorie intake had decreased by 48% from 4019 kilocalories before intervention to 2175 kilocalories after. This difference was found to be statistically significant. Total protein intake increased from a mean of 0.94 g/kg body weight to 1.47 g/kg body weight after intervention revealing a 6.38% rise and was statistically highly significant with a value of p as 0.0001.

The significant decrease in calorie intake and increase in protein intake resulted in a significant decrease in all the anthropometric parameters. The body weight showed a significant decrease of 11.5%. Similarly, the body mass index decreased by 11.6% after intervention which was statistically highly significant. The waist circumference, hip circumference, and waist-hip ratio also showed a highly significant decrease after the intervention. Findings similar to our study were also reported by Cienfuegos et al. [15] and Srivastav et al. [16]. The reason for this significant decrease in obesity can be very well attributed to the dual intervention of an increase in energy expenditure and a decrease in energy intake. A study carried out by Sindhu [17] indicates that the prevalence of obesity was 15.25% in young adults and an attempt was made to identify the role of certain factors that promote weight loss in obese young adults.

Many studies found that an increase in physical activity levels and healthy lifestyle changes in dietary patterns showed a significant change in anthropometric parameters after giving appropriate intervention to the study subjects. These findings are consistent with studies done by Stoner et al. [18], Aveyard et al. [19], and Shalitin et al. [12].

Limitations 

Although the present study covered a sizeable sample it has certain limitations which are inherent to all interventional studies. The generalizability of the study findings is limited to the study area of the present survey. Further studies on a larger sample or with a different study design may be carried out for assessing the exact amplitude of the problem as well as to establish a causal relation.

## Conclusions

Dietary modification combined with physical activity for an average of 75 minutes are the most effective short-term interventions for weight loss. The present study concludes that lifestyle modifications can reverse the trend of obesity. It is reversible and obese individuals can normalize their body mass index with appropriate interventions as was performed with the present obese study participants. A significant change can be seen in calorie intake, protein intake and all the anthropometric parameters before and after three months of intervention.
